# Supplementary material for: Understanding Internal and External Drivers Influencing the Prescribing Behaviour of Informal Healthcare Providers with Emphasis on Antibiotics in Rural India: A Qualitative Study
Source: Antibiotics (Basel). 2022 Mar 29;11(4):459. doi: 10.3390/antibiotics11040459 (PMC9029264; doi:10.3390/antibiotics11040459)
Supplement: Supplementary file 1 [file antibiotics-11-00459-s001.zip › antibiotics-1611636-supplementary.pdf]

Table S1: Topic guide

Start time of discussion:
End time of discussion:
Intro: Thank you for coming and that you also agree to participate in the study. We are talking to you since you are a health care practitioner and meet patients who are children suffering from common childhood illnesses.
The aim of this discussion is to study about the most common illnesses you encounter in children under 5 years of age, how you treat these illnesses, what treatment is given, what factors influences your treatment and what do you understand about antibiotics.
We are doing this in order to understand what factors affect in treating these illnesses in children and to find out measures which could help you while prescribing.
For your information this interview will be tape recorded, transcribed verbatim and then analyzed by team of researchers. The information recorded about you and the discussion will be kept confidential and will be deleted after the purpose of the study is achieved. You can withdraw from the study at point of time.

- Do you have any question to what I have explained till now?
- Could you please sign the consent form?

Thank you so much for listening, understanding and continuing with us for the discussion.

- Please introduce yourself by telling about your name, age, background qualification, specialty, working position, years of experience and training if any.
  (**NOTE:** A lot a number to each of the participants in the discussion to keep a track on order of their speaking while transcribing.)

Now starting with the discussion:
Q1. What kind of illnesses do you usually encounter in children below 5 years of age in your area?
- Do they vary with season and area of location?
  (To other – Do you encounter some other illnesses as more common in children below 5 years of age in your area?)

Q2. What were the illnesses you encounter in children below 5 years of age in last few days?
- Please describe what did you do? How did you treat?
- How you made the diagnosis?
- What do you understand about bacterial and viral infection?
- How did you choose on the treating medicines?

Q3. Do you always treat this illness in the similar way?
- If no, then how?
- Why did you change the treatment plan? What factors affect your treatment decision this time?

Q4. In your treatment given to the child for the illness discussed. You have given antibiotics.
- What do you understand about antibiotics?
- From where did you get this information?
- How do you decide on what antibiotics to be given and when?
- What emphasis is there on antibiotics prescription for the above discussed illness according to your knowledge and training?
- Has this changed with your increasing clinical experience?

Q5. How do you decide on the dosage given?
Q6. If after completion of the prescribed dosage, patient comes back with the same illness:
- What decision on the treatment you make at this point?
- What would make you change the treatment already given? (antibiotic treatment)
- What is the reason that your first line of treatment did not work?

Q7. Do you think that the patient and the patient's condition influence your prescribing, and if so, in what way?
Q8. How is your access to new information, updates and help, when you need it?
Q9. What do you think, why prescribing practices for the similar illnesses differ between different providers?
Q10. What do you understand about antibiotic resistance?
At the end:
If you wish to give us some advice on the measures you think would be most useful to provide you with easy access to the new information in order to improve antibiotics prescribing.
And is there anything else you would like to add before we finish.

Thank you so much for your time!
